# Improving clustering with metabolic pathway data

**DOI:** 10.1186/1471-2105-15-101

**Published:** 2014-04-10

**Authors:** Diego H Milone, Georgina Stegmayer, Mariana López, Laura Kamenetzky, Fernando Carrari

**Affiliations:** 1 Research Center for Signals, Systems and Computational Intelligence, sinc(i), FICH-UNL, CONICET, Ciudad Universitaria UNL, (3000) Santa Fe, Argentina; 2 Instituto de Biotecnología, Instituto Nacional de Tecnología Agropecuaria (IB-INTA), CONICET, PO Box 25, B1712WAA Castelar, Argentina

**Keywords:** Clustering, SOM training, Pathway data

## Abstract

**Background:**

It is a common practice in bioinformatics to validate each group returned by a clustering algorithm through manual analysis, according to *a-priori* biological knowledge. This procedure helps finding functionally related patterns to propose hypotheses for their behavior and the biological processes involved. Therefore, this knowledge is used only as a second step, after data are just clustered according to their expression patterns. Thus, it could be very useful to be able to improve the clustering of biological data by incorporating prior knowledge into the cluster formation itself, in order to enhance the biological value of the clusters.

**Results:**

A novel training algorithm for clustering is presented, which evaluates the biological internal connections of the data points while the clusters are being formed. Within this training algorithm, the calculation of distances among data points and neurons centroids includes a new term based on information from well-known metabolic pathways. The standard self-organizing map (SOM) training *versus* the biologically-inspired SOM (bSOM) training were tested with two real data sets of transcripts and metabolites from *Solanum lycopersicum* and *Arabidopsis thaliana* species. Classical data mining validation measures were used to evaluate the clustering solutions obtained by both algorithms. Moreover, a new measure that takes into account the biological connectivity of the clusters was applied. The results of bSOM show important improvements in the convergence and performance for the proposed clustering method in comparison to standard SOM training, in particular, from the application point of view.

**Conclusions:**

Analyses of the clusters obtained with bSOM indicate that including biological information during training can certainly increase the biological value of the clusters found with the proposed method. It is worth to highlight that this fact has effectively improved the results, which can simplify their further analysis.

The algorithm is available as a web-demo at http://fich.unl.edu.ar/sinc/web-demo/bsom-lite/. The source code and the data sets supporting the results of this article are available at http://sourceforge.net/projects/sourcesinc/files/bsom.

## Background

In the biology field, clustering is implemented under the guilt-by-association principle [1], that is to say, the assumption that compounds involved in a biological process behave similarly under the control of the same regulatory networks [2]. It is assumed that if a metabolic compound with unknown function varies in a similar fashion with a known metabolite from a defined metabolic pathway, it can be inferred that the unknown element is also likely to be involved in the same pathway [3]. Therefore, one cluster that groups some metabolites indicates that they can be connected within common metabolic pathways. This pathway-based approach to identify metabolic traits results in more biological information (hypothesis) that has to be tested through the design of biological experiments (wet experiments) [4]. From this perspective, it could be useful to perform a detailed inspection of the patterns inside a cluster to determine memberships to known metabolic pathways.

Due to the limitations of traditional algorithms, computational intelligence has been recently applied to bioinformatics with promising results [5,6]. For example, self-organizing maps (SOMs) [7] are a special class of neural networks that use competitive learning. SOMs can represent complex high-dimensional input patterns into a simpler low-dimensional discrete map, with prototype vectors that can be visualized in a two-dimensional lattice structure, while preserving the proximity relationships of the original data as much as possible. SOMs have been used for unsupervised clustering of transcriptome profiles [8,9] as well as metabolites [10]. For example in [11] SOM clustering was used for the analysis of *Arabidopsis thaliana*datasets, helping in the hypothesis validation of a metabolic mechanism responding to sulfur deficiency. SOMs have been recently proposed also for the integration and knowledge discovery of coordinated variations in transcriptomics and metabolomics data [12], and a software tool for SOM application has been designed to give support to the data mining task of datasets derived from different databases, providing user-friendly interface and several visualization tools easy to understand by non-expert users [13].

When evaluating a clustering solution, it is a common (and necessary) practice to validate each group returned by a clustering algorithm through manual analysis and visual inspection, according to *a-priori*biological knowledge. Traditionally, the known annotations are used only as a second step, after data have been clustered according to their variation patterns. Only those clusters in which many genes (and proteins/metabolites) are annotated within the same category (for example, the same MapMan BIN [14] or Gene Ontology (GO) terms [15]), are then selected for further analysis [16-19]. For each pattern, its annotations and memberships to well-known metabolic pathways are generally assessed. The results obtained after inspection of each cluster, by hand, may indicate functionally related patterns. Automatic pos-clustering validation proposals like “gene set enrichment analysis” [20] focus on groups of genes that share common biological function, chromosomal location or regulation. Similarly, ProteinProtein-Interactions (PPI) derived metrics can be used in combination with genomic data to validate clusters with respect to their biological relevance [21]. These metrics, however, can only be applied to clusters of genes. Recently, a biologically inspired validity measure that can be applied not only to groups of genes but also to genes and metabolites together has been proposed [22].

Actually, there is a growing interest in improving the cluster analysis of biological data by incorporating such prior basic knowledge into the clustering itself, in order to increase the biological meaning of the clusters that are subjected to later scrutiny. In the past few years, several methods have been introduced with that aim, since integrating a biological similarity measure or biological information into a clustering method can lead to the potential enhancement in the performance of the clustering, as a result of the good correlation between biological similarity and gene co-expression levels [23,24]. For example, the distance function built by [25] combines information from expression data and the proximity of the proteins in a metabolic pathway network. In [26] a similar approach is presented, where a graph is used based on the GO structure. The work of [27] proposed shrinking the distances between pairs of genes sharing a common annotation. In fact, the distance measure between two genes can be modified to be a linear combination of the similarity of their expression profiles and their functional similarity [28-30]. Moreover, a classical clustering method can be modified to work with such a newly defined metric, for example, by slicing a hierarchical clustering tree obtained from a gene dataset to get clusters that are as consistent as possible with well-known gene annotations [31]. Another example of using heterogeneous genomic data into a clustering algorithm is proposed by [32], with the aim of identifying highly correlated genes more effectively than using only expression data or a single data source. Most of these clustering methods utilize only the annotations provided by the GO ontology or its hierarchical structure through the use of similarity measures between terms. Although GO is heavily used in systems biology, redundancy and problems with stability over time have been recently indicated [33]. Besides, this information, cannot be associated to other molecular entities such as metabolites. It can be used for genes and their products only. Additionally, there are many genes that are currently unannotated and this situation is generally handled by excluding them from the analysis or by considering them as exceptional cases.

In summary, it can be anticipated that the integration of -omics measurements with additional relevant biological information is expected to improve the quality and the biological significance of unsupervised clustering. This paper proposes and illustrates this integrative principle, not only for genomic data but also for metabolic and integrated datasets. We present a novel training algorithm that combines biological similarities derived from metabolic pathways information and demonstrate that its application improves the quality of the clustering. This new approach weights the biological connectivity of the patterns (genes and/or metabolites) during training of the clustering method. This can be achieved through the use of a new term for the biological assessment of the clusters while they are being formed. The algorithm takes into account not only the classical Euclidean distance between patterns, but also a biological term assessed by means of the number of common pathways. The proposed approach was tested on a set of transcriptome and metabolome data from *Solanum lycopersicum*and *Arabidopsis thaliana*, showing improved clusters formation when using the proposed biologically inspired SOM (bSOM), in comparison to the standard SOM training (sSOM). This improvement is demonstrated by the increase of biological connections in the clusters found by bSOM and the biological analysis of the clusters found.

## Methods

In the following section we explain in detail the new biologically-inspired algorithm for SOM training. After that, the validation measures used for performance comparison among training algorithms are presented. Finally, the datasets used for SOM training are described.

### Improved SOM training using metabolic pathways

SOM clustering is based on nodes (neurons) that compete in response to a given input. Inputs are fully connected to the output nodes. Each output node corresponds to a cluster and is associated with a prototype or synaptic weight vector [34]. Given an input pattern, competition among neurons takes place, when their similarity (or distance) to the input is computed. Thus, the neurons in the output layer compete with each other, and only the closest to the input becomes activated or fired. The weight vector of this winning neuron is further moved towards (closer to) the input pattern. This competitive learning paradigm allows learning for the neuron that best matches the given input pattern and it is also known as winner-takes-all learning [35].

When competition among the neurons is complete, SOM updates not only the weight vector of the winning neuron but also a set of weights within its neighborhood, according to a neighborhood function *Λ*. This function defines the neurons that will be affected by the changes in the winning neuron. We have used the standard squared neighborhood. Thus for example, if the radius of the neighborhood is 1, all the 8 neurons in touch with the winning one will be updated as well. At the beginning of training, *Λ* has a radius equal to a quarter of the size of the map. During training, this radius is reduced linearly with training epochs, until reaching 0 (that is to say, at this point only the winning neuron is updated). The rate of the modifications at different neurons is a monotonically decreasing scalar function of the training epochs. Its form is not so important as long as its value is large at the beginning of the process, gradually reducing it to a fraction of it in successive steps [7].

The goal of SOMs is to represent complex highdimensional input patterns into a simpler low-dimensional discrete map, with prototype vectors that can be located in a two-dimensional lattice structure, while preserving the proximity relationships of the original data as much as possible [36]. SOM structures the output nodes (neurons) in such a way that nodes in closer proximity are more similar to each other than to other nodes that are farther apart. Having finished the training, input patterns are projected into the lattice, corresponding to adjacent neurons connected to each other through the neighborhood function, giving a clear topology of how the network fits into the input space [35]. In this projection, an input pattern is associated to a neuron (cluster) simply according to minimum distance to all neuron prototypes.

In Algorithm 1 we present a new algorithm for SOM training over biological datasets (bSOM). The following notation is used: *X* is the dataset formed by **x**_*ℓ*_ data samples; *Ω*_*m*_ is the set of samples that have been grouped in the cluster *m* and *W* is the set of the **w**_*m*_ centroids of the clusters. We propose the use of a combination of the classical Euclidean distance among patterns and the neurons centroids, plus an additional term that measures the internal biological connectivity of the patterns grouped in a cluster (line 7). The distance is computed using the weighted sum

(1) $$d_{\mathrm{\ell m}}=(1-\alpha )\;\varepsilon_{\mathrm{\ell m}}+\alpha \;b_{\mathrm{\ell m}},$$

where *α* is a regularization parameter that can be varied between 0 and 1 and controls the weight given to the biological distance during training; *ε*_*ℓ* *m*_=∥**x**_*ℓ*_−**w**_*m*_∥_2_ is the standard Euclidean distance between a pattern *ℓ* and a neuron prototype **w**_*m*_; and *b*_*ℓ* *m*_ is the biological contribution of a pattern *ℓ* to a cluster *m*, computed as

(2) $$b_{\mathrm{\ell m}}=\frac{\pi_{\ell \notin m}-\pi_{\ell \in m}}{\max\left\{\pi_{\ell \notin m},\pi_{\ell \in m}\right\}},$$

where *π*_*ℓ*∉*m*_ is the average number of biological connections among all the patterns clustered in the neuron *mnot including* the pattern *ℓ*; and *π*_*ℓ*∈*m*_ is the average number of biological connections among all the patterns clustered in the neuron *mincluding* the pattern *ℓ*. The average biological connections are calculated using a metabolic pathways connection matrix *ρ*, where each element *ρ*_*i* *j*_ has the number of metabolic pathways that involve both pattern in row *i* and pattern in column *j*. This is calculated by simply counting the number of pathways in common, following the same procedure for metabolites as well as for transcripts.

The biological term *b*_*ℓ* *m*_ measures how close (or distant) is a pattern *ℓ* to a neuron *m*, in terms of improvement of the average number of common pathways in that cluster. When a pattern has *b*_*ℓ* *m*_>0 with respect to neuron *m*, it means that if the pattern *ℓ* were assigned to the neuron *m*, the average number of common pathways among all the data patterns clustered in that neuron would be decreased. Instead, if *b*_*ℓ* *m*_<0, the assignment of the pattern *ℓ* to the neuron *m* would certainly increment the number of average common pathways, clearly increasing the biological value of that cluster. The parameter *α* is used to balance between the two goals: when *α*=0, *d*_*ℓ* *m*_ becomes the classical Euclidean distance and the algorithm becomes the standard SOM clustering (sSOM); and when *α*=1 the algorithm completely disregards the expression measures and groups data only according to biological connections. In principle, it cannot be stated that there is any optimum *α*, it depends on the weight that is given to the related biological information in the final analysis.

**Figure 1:**
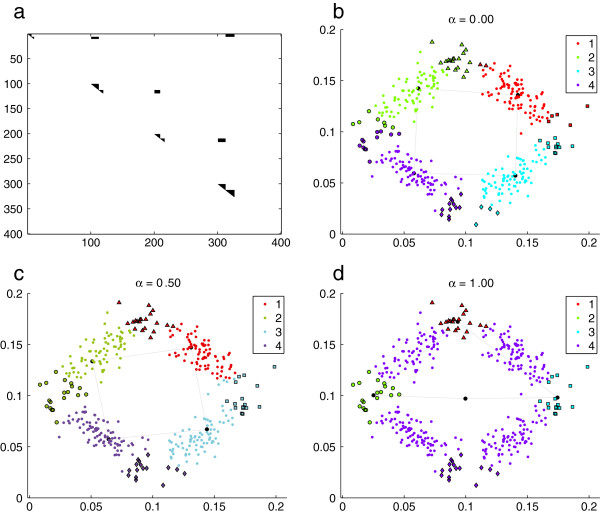
**Example of SOM training using metabolic pathways (bSOM) with an artificial data set.a**) *ρ* matrix; **b**) *α*=0.00; **c**) *α*=0.50; **d**) *α*=1.00 (the two dimensions in this simplified example could represent measures for two different treatments in real data). Each cluster found by the algorithm is indicated with a different color (red, green, cyan and purple). Groups of biologically related points are indicated with different markers (squares, diamonds, circles and triangles).

An artificial “toy-example” data set has been used to illustrate the new algorithm. It is shown in Figure 1. The set consists of four groups of 100 data points each, following Gaussian distributions. In Figure 1a) the *ρ* matrix corresponding to this data set is shown. For simplicity purposes, the matrix is stored as upper triangular. The color of the pixels indicate the existence of biological connections among elements. In this artificial example, four groups biologically connected can be distinguished by looking at the main diagonal (black pixels). The rest of the data points do not have pathways in common (white pixels). In the remaining sub-figures, the data points distribution is shown, as well as the groups and the centroids of each cluster (black dots) obtained for *n*=2 (that is, 4 neurons). Each cluster found by the algorithm is indicated with a different color. The points located at the extremes of the groups are biologically related among them, as indicated with different markers (squares, diamonds, circles and triangles) which correspond to the four groups of high biological connections present in the *ρ* matrix. In Figure 1b), *α*=0.00 is used (equivalent to sSOM). It can be seen that the neurons centroids are located approximately in the euclidean center of each distribution. In the case of *α*=0.50, shown in Figure 1c), the Euclidean distance as well as the biological connections are used to form the clusters. It can be noticed that the centroids here have been moved in order to group in the same cluster some of the patterns biologically connected. At the extreme, with *α*=1.00 in Figure 1d), only the groups that have common pathways among their elements form a cluster and determine the centroids location. As it can be clearly seen from this example, when *α* is increased the biological connections among elements increase their direct influence on the clustering results.

### Validation measures

After the application of an unsupervised mining technique, it is quite difficult to validate the obtained results. A set of objective measures can be used to quantify the quality of the clusters obtained by different available methods [34]. A new kind of biological measure is presented as well, that evaluates the metabolic connections existing in the clustering partition found. The work of [37] presents a summary of different types of validation measures that can be used to qualify a clustering solution. In this study we have used:

#### Compactness

It measures intracluster compactness or homogeneity as ${\bar{C}}_{j}=1/|\Omega_{j}|\underset{\forall x_{i}\in \Omega_{j}}{\sum }∥x_{i}-{\text{w}}_{j}{∥}_{2}$, For a global measure of compactness, the average over all *k* clusters is calculated as $\bar{C}=1/k\underset{j}{\sum }{\bar{C}}_{j}$. Values of $\bar{C}$ close to 0 indicate more compact clusters.

#### Separation

It quantifies the degree of separation between individual clusters, measuring the mean Euclidean distance among cluster centroids as $\bar{S}=2/\;\left(k^{2}-k\right)\overset{k}{\underset{i=1}{\sum }}\overset{k}{\underset{j=i+1}{\sum }}∥{\text{w}}_{i}-{\text{w}}_{j}{∥}_{2}$, where $\bar{S}$ close to 1 indicates more separated clusters.

#### Davies-Bouldin index

This is a combination of the previous two measures and a popular metric for evaluating clustering algorithms [38]. *DB* index is a function of the ratio of the sum of within-cluster scatter to between-cluster separation. This is an indication of clusters overlap, therefore *DB* close to 0 indicates that the clusters are compact and far from each other.

#### Dunn index

It combines dissimilarity between clusters and their diameters, based on the idea of identifying cluster sets that are compact and well separated. *D* index measures inter-cluster distances (separation) over intra-cluster distances (compactness). If a clustering partition contains well-separated clusters, the distances among them are usually large and their diameter is expected to be small. Therefore, a larger *D* value means better cluster configuration.

#### Biological internal connectivity

For the evaluation of the clusters from the viewpoint of their biological meaning, we use a measure defined as follows:

(3) $$\bar{P}=-\log\left(\frac{1}{k}\overset{k}{\underset{m=1}{\sum }}\frac{p_{m}}{p_{m*}}\right),$$

where

pm=1+∑∀i/xi∈Ωm∀j/xj∈Ωmj≠iρij

is the number of common pathways among patterns grouped in cluster *m*, with *ρ*_*i* *j*_ the number of pathways in common between patterns *i* and *j*, and

pm∗=1+∑∀i/xi∈Ωm∀j≠iρij,

is the number of all the possible shared pathways among patterns grouped in cluster *m* and any other pattern in the dataset. A $\bar{P}$ value close to 0 indicates more biologically significant clusters. For this measure, non empty and annotated clusters are taken into account.

#### Global Measure for Linked Clustering (GMLC)

For evaluating both coherence and biological significance of clusters found over biological datasets, we have used the *G* measure which is a biologically-inspired validity measure for comparison of clustering methods over metabolic datasets [22]. It is defined as the sum of $\overset{ˇ}{H}$, which is a measure of the flatness of the distribution of patterns along clusters, $\bar{\Gamma }$ that indicates if the data samples have been coherently grouped when having a sign-inverted value, and $\bar{P}$ which evaluated biological internal connectivity, as previously explained.

### Datasets

In this subsection, the datasets used for SOM training are described. The Kyoto Encyclopedia of Genes and Genomes (KEGG) [39,40] pathway database was used for calculation of the biological connectivity. All pathways in which the measured elements participated have been considered.

#### *Solanum lycopersicum* dataset

The first biological dataset used in this paper involves metabolic and transcriptional profiles from Introgression Lines (ILs) of *Solanum lycopersicum*. The ILs harbor, at certain chromosomes segments, introgressed portions of the wild species (*Solanum pennellii*). After log-transforming the expression values over the entire dataset, genes with no significant change were discarded from further analysis. As a result of the pre-processing and selection steps, 1159 genes were selected. The metabolic data were obtained analyzing polar extracts of tomato fruits, through Gas Chromatography coupled to Mass Spectrometry (GC-MS). The metabolite profiling technique used allows the identification of approximately 80 primary metabolic compounds. For each metabolite in each IL, the log ratio of the mean of the replicates was calculated. In the selection step only 70 metabolites (having log ratio greater than 0.1) were kept for data integration and cluster analysis. Further details on data selection can be found on [12]. This data set has a size of 1229 data points.

#### *Arabidopsis thaliana* dataset

The second biological dataset comprises primary metabolites and transcripts measured in *Arabidopsis thaliana* leaves. The integrated analysis of this data is aimed at studying the effects of the cold on circadian regulated genes in this plant [41]. In this study we included metabolites and transcripts under light-dark cycles at two control temperatures (20°C and 4°C). Genes involved in diurnal cycle and cold-stress responses were selected for further study. More details on how the data were processed, filtered and normalized can be found in [41]. A total of 1549 genes and 51 metabolites were used in the integrated analysis, resulting in a total of 1600 data patterns.

## Results and discussion

**Figure 2:**
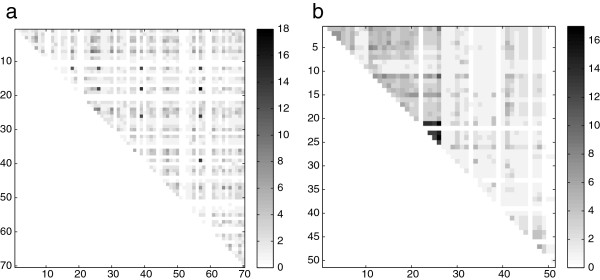
**Biological internal connectivity of data sets.** Corresponding *ρ* matrix for: **a**) *Solanum lycopersicum*, **b**) *Arabidopsis thaliana* data sets. The intensity in the gray scale indicates a higher connection value.

This section presents the results obtained from the application of the new biologically-inspired training algorithm (bSOM), in comparison to the standard training (sSOM). For a preliminary assessment, only the metabolic profiles of each data set were used since all metabolites have information associated to metabolic pathways. The corresponding *ρ* matrix for *Solanum lycopersicum* and *Arabidopsis thaliana* datasets, respectively, are shown in Figure 2. The intensity in the color scale indicates a higher connection value. It can be seen that most of the data points have metabolic pathways in common (there is a very low number of white pixels). There are just few points highly connected (black pixels), but most of the data points have a moderate number of common pathways. For this reduced subset, a map size of 6×6 neurons was used. This allowed us to easily evaluate whether there was an improvement in the biological connections of the clusters found by the new bSOM in comparison to sSOM. The *α* parameter that weights the biological distance has been varied in the range [ 0.00,0.25,0.50,0.75].

Table 1 shows the results of the comparison of both training algorithms over the two biological datasets, for the validation measures presented in the Validation measures Section. For each measure, a triangle up or down is shown in order to easily indicate whether the best expected index value should have a high or a low value. Compactness and average separation of the clusters are slightly worsened when using bSOM in comparison to sSOM in both data sets. This is due to the fact that these measures are highly dependant on the Euclidean distance and at higher *α* the biological term has a stronger influence on the assignment of patterns to clusters than the Euclidean distance. The *DB* measure does not improve when using bSOM in any case, on the contrary, it gets worst scores. This can be expected since, in fact, this measure is designed to better qualify well-separated and highly compact clusters (in the Euclidean sense) and, as explained above, compactness and separation are worsened as *α* increases. Similar behavior has the Dunn index for the first data set, although improving with bSOM and *α*=0.5 and *α*=0.75 for the second dataset. Although these two measures are a combination of compactness and separation into one single index (Dunn qualifies clusters taking into account the same general criteria as DB) they present contradictory results. While DB uses the Euclidean distance of data to centroids in a direct way, Dunn measures the distance to the global centroid of all data and not between data and each cluster centroid.

**Table 1:** **Validation measures for SOM training: standard (sSOM) *versus * biological (bSOM) for metabolic datasets**

	**sSOM**		**bSOM**	
***α*** **→**	**0.00**	**0.25**	**0.50**	**0.75**
*Solanum lycopersicum*				
${\bar{C}}^{▿}$	0.65	0.69	0.73	0.79
${\bar{S}}^{▵}$	0.66	0.65	0.59	0.49
$DB^{▿}$	9.56	31.00	13.90	19.05
$D^{▵}$	0.59	0.40	0.40	0.37
${\bar{P}}^{▿}$	3.58	2.74	2.58	2.08
$G^{▿}$	0.87	0.90	0.83	0.87
*Arabidopsis thaliana*				
${\bar{C}}^{▿}$	0.48	0.55	0.64	0.65
${\bar{S}}^{▵}$	0.81	0.79	0.79	0.71
$DB^{▿}$	10.45	8.86	490	60.66
$D^{▵}$	0.32	0.24	0.50	0.54
${\bar{P}}^{▿}$	3.84	2.93	2.17	1.56
$G^{▿}$	0.65	0.67	0.52	0.48

With respect to measures that take into account the biological information associated to the clusters obtained, considering the $\bar{P}$ measure, it is clearly and consistently improved as *α* increases for the proposed algorithm when compared to sSOM, for all configurations and both datasets. As can be expected, at low *α* the improvement is not so important but when *α* increases, clusters are more biologically connected which is directly reflected by this measure, reaching the best possible result for this index at the maximum *α* here considered. The significance of these results has been statistically tested by performing 100 re-samplings of 90% of the metabolites in both datasets, for all the methods (sSOM vs. bSOM with different *α*). An ANOVA was performed to test the null hypothesis in which the difference among the clustering results for the biological connectivity measure ($\bar{P}$) with different training methods is not significant. The analysis revealed that the results in the table show significant differences (*p*<0.001). Finally, the *G* measure, which evaluates in a single index not only clusters quality but also their biological content, remains almost unchanged or even improves. For the first data set, *G* has almost the same value in all configurations. As *α* is increased on bSOM, *G* values improve for the second data set, even at maximum *α*. In general, it can be stated that while a balance between homogeneity and coherence is maintained, an improvement in the biological connectivity of the clusters can be achieved.

Table 2 shows the results of the comparison of both training algorithms over the two full biological datasets (transcripts and metabolites). The Gap Statistic [42], intended to estimate adequate cluster numbers from a dataset [43], was used to select the number of clusters for the comparisons among methods. The selected map size was 10×10 neurons. Comparisons between sSOM and other clustering algorithms for the datasets used in this study have already been done in [22]. It is worth to highlight the fact that, although all metabolites were annotated, only a low proportion of the genes (approximately 10%) were associated to metabolic pathways in the KEGG database. In this case, with so many clusters without related biological information, one should expect that it will be very hard to enhance the results, even using high *α* values. However, the results obtained in both cases show that bSOM can work well even in this situation, improving the biological connections of the clusters. Considering the classical data mining measures in Table 2, the results do not vary significantly between methods and configurations tested. For example, compactness as well as separation remain almost unchanged in all cases. The *DB* index is particularly influenced in the case of large *α* since the Euclidean distance is almost disregarded for grouping data points and thus the clusters get closer and larger, which is highly penalized by this measure. The Dunn index is slightly worsened in most cases, improving only in one case with a large *α* in the first data set.

**Table 2:** Validation measures for SOM training: standard (sSOM) and biological (bSOM) for the full datasets

	**sSOM**		**bSOM**	
***α*** **→**	**0.00**	**0.25**	**0.50**	**0.75**
*Solanum lycopersicum*				
${\bar{C}}^{▿}$	0.79	0.80	0.80	0.81
${\bar{S}}^{▵}$	0.68	0.67	0.66	0.64
$DB^{▿}$	8.80	9.07	9.12	10.64
$D^{▵}$	0.18	0.14	0.13	0.26
${\bar{P}}^{▿}$	3.32	2.65	2.38	1.80
$G^{▿}$	1.09	0.63	0.59	0.52
*Arabidopsis thaliana*				
${\bar{C}}^{▿}$	0.51	0.52	0.51	0.51
${\bar{S}}^{▵}$	1.00	1.00	1.00	1.00
$DB^{▿}$	13.30	12.02	10.35	12.19
$D^{▵}$	0.16	0.15	0.16	0.13
${\bar{P}}^{▿}$	3.13	3.10	2.80	2.00
$G^{▿}$	0.68	0.41	0.43	0.32

Taking into consideration now only the measures that evaluate the biological quality of the solutions ($\bar{P}$ and *G*), both present better results and it can be stated that, in general, the biological connectivity of the clusters is really improved when using bSOM compared to sSOM, in both datasets. The biological connectivity of the clusters is effectively improved when using bSOM in comparison to sSOM, which is even achieved when both distances (Euclidean and biological) are equally considered (*α*=0.5). The *G* measure also consistently obtains better scores when *α* increases, in all configurations tested for each map. This means that enhanced clustering results can be achieved when using bSOM rather than sSOM, not only with respect to clusters quality but also from a biological point of view.

For the full Arabidopsis dataset, we have also calculated the biological homogeneity index (BHI) [44] for sSOM and bSOM, which measures how homogeneous are biologically the clusters obtained. BHI evaluates if genes in the same cluster are also part of the same functional classes according to GO annotations. The BHI score obtained for sSOM was 6.49%. For bSOM with the same *α* values reported in Table 2, the BHI scores were 6.57, 6.68 and 7.53%. As can be seen, this independent measure also indicates that better biological clusters can be obtained with the proposed algorithm.

Finally, to show an illustrative example of how bSOM obtains better clustering results from a biological point of view, a pathways analysis and validation has been performed over neurons selected at random from a SOM map on the first data set. Table 3 shows comparative results regarding the data points that where clustered in the neurons by both algorithms and the *Solanum lycopersicum* dataset. The full statistics for all clusters in both datasets have been presented in the previous tables.

**Table 3:** Detail of patterns and common pathways for sSOM vs. bSOM

**Algorithm** →	**sSOM**	**bSOM**
Cluster A	Serine	Serine
	Threonine	Threonine
	Valine	Valine
	Glycine	Isoleucine
	Lysine	
Common	ko00260, ko00290	ko00260, ko00290
pathways	ko00970, map1060	ko00970, map1060
	ko02010	ko02010
	ko00460	ko00966
Cluster B	Arginine	Arginine
	*β*-alanine	Glycine
	GABA	Lysine
Common	ko00330, ko00410	ko00310, ko00970
pathways	ko04080	map1060, map1064
		ko02010
Cluster C	LE31F17	LE31F17
	LE30O12 ^∗^	LE16F20
	LE26F02 ^∗^	
Common	-	ko00052
pathways		ko00511, ko00531
		ko00600, ko00604
Cluster D	Sucrose	Sucrose
	Aspartate	Glutamate
	5oxoproline	Proline
		LE23B16 ^∗^
		LE23N08 ^∗^
Common	ko02010	ko02010
pathways		ko00330, ko00970

^*^does not participate in a well-known pathway.

**Gene transcript codes.** LE31F17: beta-galactosidase (GB acc# AAC25984); LE16F20: beta-galactosidase (GB acc# AAC25984); LE30O12: no data; LE26F02: component of oligomeric golgi complex, putative (GB acc# XP_00251994); LE23B16: CDPK-related protein kinase (GB acc# AAZ83348); LE23N08: no data.

**Pathway codes.** ko00260: Glycine, serine and threonine metabolism; ko00290: Valine, leucine and isoleucine biosynthesis; ko00970: Aminoacyl-tRNA biosynthesis; map01060: Biosynthesis of plant secondary metabolites; ko02010: ABC transporters; ko00460: Cyanoamino acid metabolism; ko00966: Glucosinolate biosynthesis; ko00330: Arginine and proline metabolism; ko00410: beta-Alanine metabolism; ko04080: Neuroactive ligand-receptor interaction; ko00310: Lysine degradation; map01064: Biosynthesis of alkaloids derived from ornithine, lysine and nicotinic acid; ko00052: Galactose metabolism; ko00511: Other glycan degradation; ko00531: Glycosaminoglycan degradation; ko00600: Sphingolipid metabolism; ko00604: Glycosphingolipid biosynthesis.

From a quantitative point of view, it can be seen that in general bSOM can increase the number of common pathways in the clusters for the same number of elements. In particular, in Cluster A the number of common pathways among cluster elements is maintained, although bSOM can achieve that result with less cluster elements. In Cluster B, for the same number of elements a higher number of common metabolic pathways was obtained. In Cluster C, a better grouping allows finding common biological information, which could not be achieved by using the standard training algorithm. Finally, cluster D exemplifies how, for the same number of elements with related biological information in a cluster, more common pathways can be found by bSOM (note that although the cluster found by bSOM has 5 elements, only 3 of them participate in known pathways).

The previous examples suggest that bSOM is able to better group the amino acids *glycine*, *serine*, *threonine*, *valine*, *leucine*, *isoleucine*, *lysine* and *arginine* within clusters considering the number of biochemical pathways they are involved in. For instance, bSOM grouped *serine*, *threonine*, *valine* and *isoleucine* within cluster A and *glycine*, *arginine* and *lysine* in a separate cluster (B). In this case, bSOM takes account of the possibility that co-variation of *valine* and *isoleucine* can also be affected by their degradative pathway (ko00280). Another example of the usefulness of bSOM is given by clusters C and D. In the first case, bSOM grouped two transcripts which both encode for *beta-galactosidase* precursor. It is somehow here expectable either because they are derived from the same gene or from different *loci*. In cluster D, *glutamate*, *proline* and *sucrose* grouped together with two transcripts. One of these transcripts (LE23B16) encodes a putative calcium-dependent protein kinase (CDPK). Although the exact mechanism by which this protein could be related to the variation of the above-mentioned metabolites is not known, the role of different CDPKs in the control of primary plant metabolism is well documented [45].

## Conclusions

In this paper we presented a new training algorithm for self-organizing maps (bSOM) over biological datasets. A new biologically-inspired term, considering common pathways, is added in the calculation of the distances among data points and neurons centroids. This term evaluates the internal connections of the data samples in terms of their belonging to known pathways. The proposed training algorithm was tested in two datasets involving *Solanum lycopersicum* and *Arabidopsis thaliana* transcripts and metabolites. Classical data mining validation measures were used to qualify the clustering solutions obtained when using both algorithms, as well as a new measure that takes into account biological significance of the clusters found. The new algorithm showed important improvements in performance in comparison to standard SOM training. It is worth to highlight the fact that the inclusion of biological information implicitly during training has effectively improved the results. This would certainly increase the biological value of the clusters found and would simplify their further analysis. Future work will involve the expansion of the range of additional biological sources that could be used in combination with clustering algorithms.

## Availability

•Project name: bSOM.

•Web-demo: http://fich.unl.edu.ar/sinc/web-demo/bsom-lite/

•Source code and data sets: http://sourceforge.net/projects/sourcesinc/files/bsom

•License: opensource, free for academic use.

## Competing interests

The authors declare no competing interests.

## Authors contributions

DM and GS proposed and implemented the clustering algorithm, and wrote the manuscript. ML, LK and FC have contributed with motivations and useful discussions, provided the case study dataset and revise the manuscript. All authors read and approved the final manuscript.
